# Low genetic diversity despite multiple introductions of the invasive plant species *Impatiens glandulifera* in Europe

**DOI:** 10.1186/s12863-015-0242-8

**Published:** 2015-08-20

**Authors:** Jenny Hagenblad, Jennifer Hülskötter, Kamal Prasad Acharya, Jörg Brunet, Olivier Chabrerie, Sara A. O. Cousins, Pervaiz A. Dar, Martin Diekmann, Pieter De Frenne, Martin Hermy, Aurélien Jamoneau, Annette Kolb, Isgard Lemke, Jan Plue, Zafar A. Reshi, Bente Jessen Graae

**Affiliations:** Norwegian University of Science and Technology, Department of Biology, NO-7491 Trondheim, Norway; IFM – Biology, Linköping University, SE-581 83 Linköping, Sweden; University of Applied Sciences Bremen, DE-28199 Bremen, Germany; Swedish University of Agricultural Sciences, Southern Swedish Forest Research Centre, SE-230 53 Alnarp, Sweden; Plant Biodiversity Lab, FRE 3498 CNRS, Université de Picardie Jules Verne, FR-80037 Amiens, Cedex France; Department of Physical Geography and Quaternary Geology, Stockholm University, SE-106 91 Stockholm, Sweden; Department of Botany, University of Kashmir, Srinagar – 190006, Jammu & Kashmir, India; Vegetation Ecology and Conservation Biology, Institute of Ecology, University of Bremen, DE-28359 Bremen, Germany; Forest & Nature Lab, Ghent University, BE-9090 Melle Gontrode, Belgium; Division Forest, Nature and Landscape, University of Leuven, BE-3001 Leuven, Belgium

**Keywords:** SSRs, Colonisation events, Exotic species, Molecular diversity, Weeds

## Abstract

**Background:**

Invasive species can be a major threat to native biodiversity and the number of invasive plant species is increasing across the globe. Population genetic studies of invasive species can provide key insights into their invasion history and ensuing evolution, but also for their control. Here we genetically characterise populations of *Impatiens glandulifera*, an invasive plant in Europe that can have a major impact on native plant communities. We compared populations from the species’ native range in Kashmir, India, to those in its invaded range, along a latitudinal gradient in Europe. For comparison, the results from 39 other studies of genetic diversity in invasive species were collated.

**Results:**

Our results suggest that *I. glandulifera* was established in the wild in Europe at least twice, from an area outside of our Kashmir study area. Our results further revealed that the genetic diversity in invasive populations of *I. glandulifera* is unusually low compared to native populations, in particular when compared to other invasive species. Genetic drift rather than mutation seems to have played a role in differentiating populations in Europe. We find evidence of limitations to local gene flow after introduction to Europe, but somewhat less restrictions in the native range. *I. glandulifera* populations with significant inbreeding were only found in the species’ native range and invasive species in general showed no increase in inbreeding upon leaving their native ranges. In Europe we detect cases of migration between distantly located populations. Human activities therefore seem to, at least partially, have facilitated not only introductions, but also further spread of *I. glandulifera* across Europe.

**Conclusions:**

Although multiple introductions will facilitate the retention of genetic diversity in invasive ranges, widespread invasive species can remain genetically relatively invariant also after multiple introductions. Phenotypic plasticity may therefore be an important component of the successful spread of *Impatiens glandulifera* across Europe.

**Electronic supplementary material:**

The online version of this article (doi:10.1186/s12863-015-0242-8) contains supplementary material, which is available to authorized users.

## Background

Invasive plant species are becoming increasingly common and can threaten biodiversity across the world [31]. Apart from being of biological importance – frequently having a negative effect on local plant communities [56, 58, 99] – invasive species also provide particular opportunities to study ecological and evolutionary processes [39]. Being just a subset of the species-wide gene pool, possibly suffering severe loss of genetic diversity upon the invasion [4, 66], they are nonetheless able to thrive in a novel environment and thereby provide useful study systems for responses to rapid environmental changes [21, 39].

The successful invasiveness of some species in spite of low genetic diversity is commonly referred to as the genetic paradox of invasive species [28]. It has, however, been shown that high genetic diversity is not a prerequisite for an invasive species to be successful [21] and some studies suggest phenotypic plasticity is instrumental for invasiveness [52, 72]. Others instead stress the importance of rapid evolutionary responses [11, 22, 53]. Molecular population genetics can be instrumental in exploring the importance of genetic components of invasiveness [51]. For example, although loss of genetic diversity is expected upon colonisation of new areas, it has been suggested that high genetic diversity, resulting from multiple introductions, could be what allows a species to become invasive [69].

Phylogeographic analysis of intraspecific genetic variation can be used to explore the migration history of a species, including species that have recently colonized an area (e.g. [41, 87] and references therein). For invasive species, phylogeographic analyses can provide information about the source population(s) in an invader’s native range, as well as elucidate patterns of spread within the species’ novel range (e.g. [68, 84, 108]). Additionally, phylogeographic patterns and the distribution of genetic diversity within and between populations can shed light on human facilitation of spread and thus aid in developing suitable management strategies [101].

*Impatiens glandulifera* Royle (Balsaminaceae), the Himalayan Balsam, is an invasive species in Europe (e.g. [18, 80]), North America and New Zealand [96, 104] with the ability to outcompete native species, particularly in riparian habitats [6, 40]. It is pollinated by insects but can also self-pollinate [80]. Dehiscence of the seed capsule spreads seeds up to a distance of 5 m while long-distance dispersal is primarily carried out by man or water currents [6]. Being an annual species it can, upon senescence, leave riverbanks exposed to winter erosion and during the growth season its roots can block and threaten land drainage schemes [80].

**Fig. 1:**
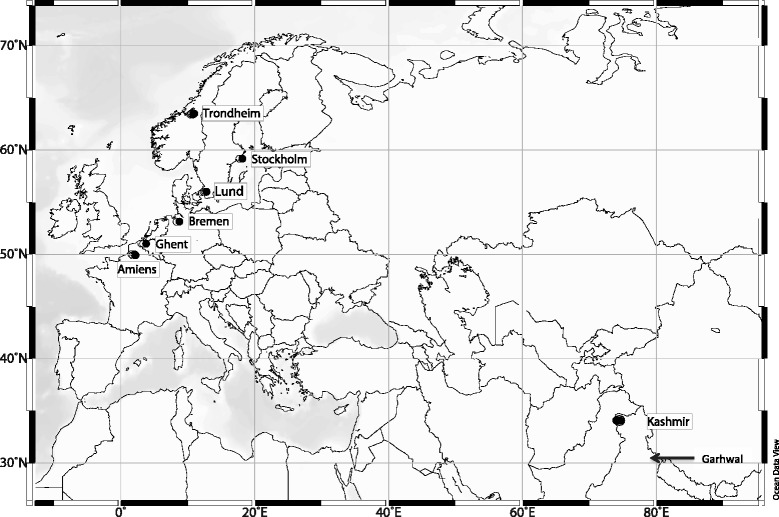
Map showing the location of the sampled municipalities of *Impatiens glandulifera* and of Garhwal, illustrating the native range of the species

In its native range *I. glandulifera* grows at altitudes of 2000 – 4000 m a.s.l. from Kashmir to Garhwal in the Northern Indian state of Uttarakhand [6, 75] (Fig. 1). The first documented European introduction of *I. glandulifera* was from Kashmir to the British Isles in 1839, where it was initially grown in the Kew Gardens [6, 15, 57]. Originally an ornamental garden flower, it was first recorded as a naturalised plant in 1855 [9]. During the 19^th^ and 20^th^ century the species gradually spread across the continent [9, 33, 37, 47, 67, 73, 80, 95, 97]. The increasingly more northern reports suggest spread may have happened in a step-by-step fashion from the range frontier, which, if true, should be evident through decreasing genetic diversity in more northern latitudes. The species is now widespread in Europe and found up to 64° N [5]. Seeds and seedlings have been brought to Europe on several occasions [47], but it is not known from which introduction(s) *I. glandulifera* populations presently found in Europe descend.

Most studies on *I. glandulifera* so far have described the spread of the species on a local or countrywide scale [33, 80], or have tried to elucidate the mechanism for its invasiveness [79, 80, 90]. In addition, differences in growth and phenology of *I. glandulifera* have been shown to be correlated with latitudinal origin, suggesting adaptation to the length of the growing season [46]. Recently, the genetic diversity of *I. glandulifera* on a local or countrywide scale has been described for British [78, 100], Lithuanian [110] and Finnish [64] populations. To our knowledge, however, there have been no population genetic studies sampling *I. glandulifera* across a larger part of its European distribution.

Here we assess both local and more large-scale patterns of genetic diversity in *I. glandulifera* by characterising the molecular genetics of populations both from the species’ native range in Kashmir (India) and the introduced range within Europe across a large part of the species’ invaded north – south distribution. The main aims of our study were 1) to investigate the number of introductions into Western Europe, 2) to compare the genetic diversity of the species and its distribution in the invaded and native range, 3) to explore the importance of evolutionary forces, in particular gene flow, between populations in shaping the distribution of genetic diversity in the invasive range and 4) to compare our results with general population genetic patterns in invasive species.

## Results

### Genotyping success and presence of null alleles

A final dataset of 378 individuals genotyped for nine markers was used to explore the population genetics of *I. glandulifera*. Originally individuals from 10 populations, some located within the same municipality, along the species’ north – south distribution in Europe were genotyped for eleven microsatellite markers and compared with individuals from three populations from Kashmir in the species’ native range (Table 1, Fig. 1). The locus IGNSSR103 failed to amplify and was therefore excluded from further analysis. The locus IGNSSR106 (14 % successfully amplifying individuals) and one individual each from the populations Amiens1 and Kashmir1 with < 30 % successfully amplifying markers were also removed due to poor success rate, leaving the final dataset to be used for further analysis.

**Table 1:** Description of the 13 studied populations of *Impatiens glandulifera*. Information about location, number of individuals studied, number of alleles found, expected heterozygosity under Hardy-Weinberg equilibrium (h), observed heterozygosity (H_O_) and inbreeding coefficient (F_IS_)

Population	Country	Latitude (°N)	Longitude (°E)	Number of individuals genotyped	Number of alleles found	Number of private alleles	h^a^	H_O_^a^	F_IS_
Amiens1	France	49.922	2.229	30^b^	22	0	0.226	0.152	0.002
Amiens2	France	50.014	2.036	30	15	0	0.154	0.157	−0.099
All Amiens						0			
Ghent	Belgium	51.010	3.794	30	17	1	0.237	0.195	−0.252
Bremen	Germany	53.130	8.786	30	20	2	0.254	0.280	−0.225
Lund1	Sweden	55.994	12.800	30	19	0	0.136	0.089	0.070
Lund2	Sweden	55.977	12.820	30	19	1	0.202	0.148	0.068
All Lund						1			
Stockholm	Sweden	59.163	18.169	30	19	0	0.265	0.246	−0.371
Trondheim1	Norway	63.479	10.999	30	16	0	0.225	0.172	−0.079
Trondheim2	Norway	63.477	10.964	30	10	0	0.067	0.115	−0.938
Trondheim3	Norway	63.413	10.809	30	16	0	0.154	0.107	0.178
All Trondheim						0			
Kashmir1	India	34.076	74.480	20^b,c^	49	1	0.665	0.458	0.126*
Kashmir2	India	34.087	74.527	30	59	11	0.599	0.361	0.224***
Kashmir3	India	34.090	74.547	30	59	8	0.623	0.432	−0.008
All Kashmir						43			

* *p* < 0.05

*** *p* < 0.001

^a^Average across markers

^b^One individual removed before data analyses due to low success rate in genotyping

^c^Only 20 individuals could be analysed due to fungal infection on the leaves of some of the individuals

Two hundred thirty-eight marker genotypes with poor quality chromatograms were genotyped a second time. Of these, the majority (87 %) yielded identical genotypes upon repeated scoring. Genotyping error rate was not estimated for samples with high quality chromatogram markers, but is expected to have been considerably lower than for low quality chromatogram markers. In 75 out of 117 marker - population combinations the frequency of null alleles was estimated to be less than 5 % suggesting that null alleles were not a predominant property in most populations (Additional file 1). The Kashmir populations typically had a null allele frequency of 20 % or higher for more markers than the European populations.

### *Distribution of genetic diversity in* I. glandulifera

After Bonferroni correction, markers deviating from Hardy-Weinberg Equilibrium (HWE) were found in all populations (Additional file 1). Some pairs of loci showed significant linkage disequilibrium (LD) after Bonferroni correction (Additional file 2). However, only two of the pairs of loci were in significant LD in more than one of the 13 populations studied.

Genetic diversity measures within European, and to a lesser extent Kashmir, populations varied markedly. Within Europe, none of the measures of diversity (Table 1) were significantly correlated with latitude of origin (for all measures *p* > 0.1). Instead populations with both comparatively high and low diversity measures could be found both among more northern and southern populations. Trondheim2, one of the northernmost populations, did, however, stand out as having the fewest number of alleles, lowest expected heterozygosity and most highly negative inbreeding coefficient of all populations (Table 1).

Both the average within-population genetic diversity (Europe: 0.210, Kashmir: 0.629) and the total genetic diversity (Europe: 0.351, Kashmir: 0.779) were lower in Europe than in Kashmir (Additional file 3, within-population diversity: *t*-test *p* < 0.001; total population diversity: Wilcoxon rank sum test *p* < 0.001). The number of alleles (Table 1) was significantly higher (Wilcoxon rank sum test *p* < 0.05) in Kashmir populations (total number of alleles 81, mean per population and locus 6.2) than in European (total number of alleles 44, mean per population and locus 1.9) as were the number of private alleles (Kashmir populations mean 6.19, European populations mean 1.92; Wilcoxon rank sum test p < 0.05). The inbreeding coefficient was significantly higher in Kashmir than in Europe (*t*-test p < 0.05) and two of the Kashmir populations (1 and 3), but none of the European populations, had significant inbreeding coefficients when calculated across all loci (Table 1).

### *Latitudinal genetic structuring in European* I. glandulifera

**Fig. 2:**
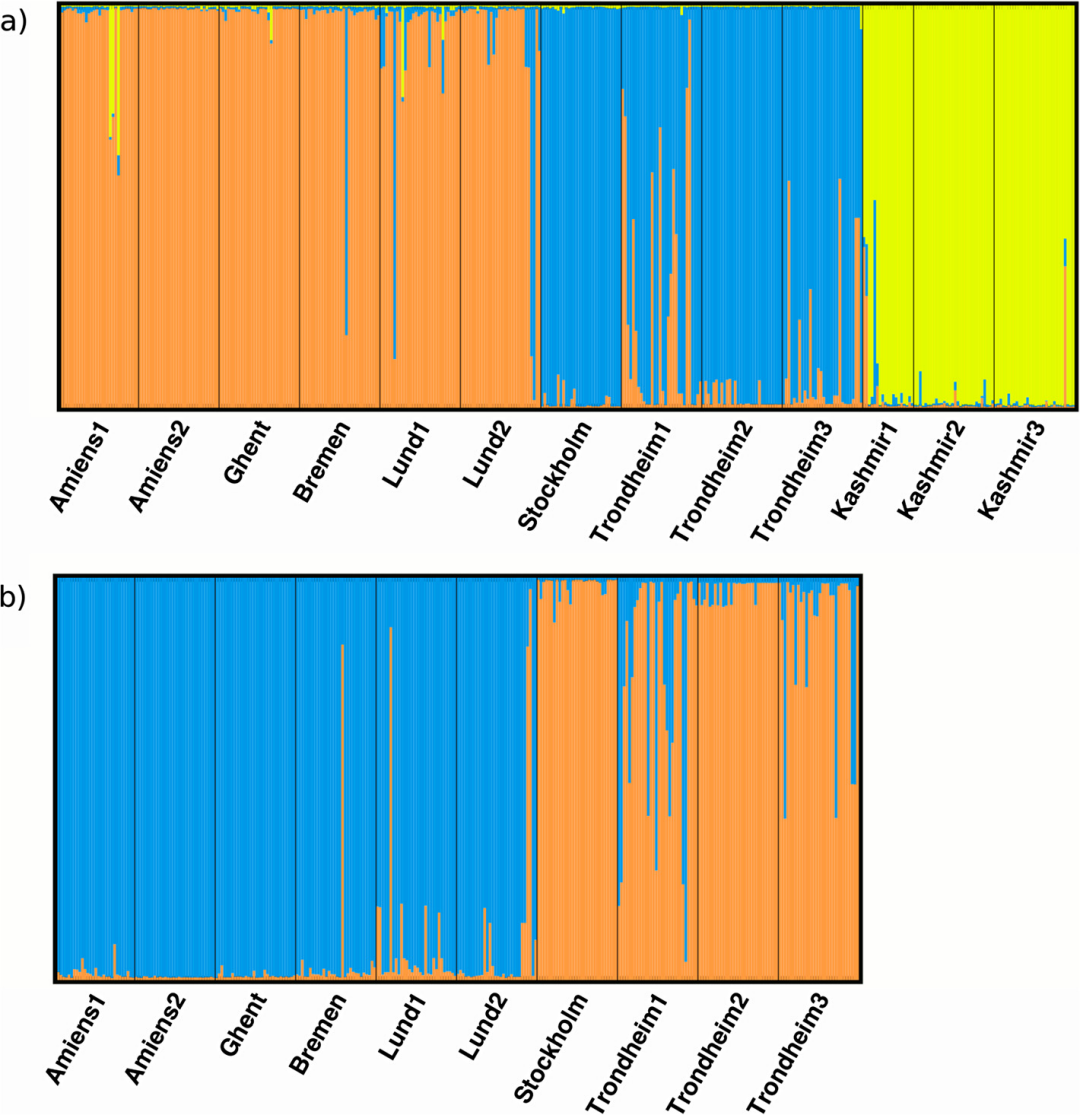
Results of the STRUCTURE analysis under the admixture model. Each individual is represented by a vertical line, with different colours corresponding to the different clusters to which a given individual has been assigned, and with the height of each colour corresponding to the amount of the genetic diversity assigned to that cluster. Results of analysis for **a**) full data set at K = 3, **b**) European individuals at K = 2

In the STRUCTURE analysis of the full dataset ΔK suggested K = 3 (ΔK = 27413) as the number of clusters best describing the data (Additional file 4). This was also the level of clustering with the highest repeatability between runs according to CLUMPP H values (H = 0.996, Additional file 4). At this level one cluster contained the Kashmir populations (with the exception of Stockholm already separated from the European populations at K = 2, ΔK = 8145.4, H = 0.985), another the more southern European populations (Amiens1, Amiens2, Ghent, Bremen, Lund1 and Lund2) and a final cluster the northern European populations (Stockholm, Trondheim1 – 3) (Fig. 2a).

In the STRUCTURE analysis of only European individuals ΔK suggested that the data were best described by two clusters (ΔK = 17208), also the number of clusters with the highest repeatability between runs according to CLUMPP H values (0.997), with the second highest ΔK and CLUMMP H values for the K = 4 model (ΔK = 1951.6, H = 0.984, Additional file 4). At K = 2 the clusters corresponded to the north – south clustering observed in the full data set (Fig. 2b). The four-cluster model additionally had a cluster containing only the Stockholm population and a cluster consisting primarily of the individuals from Amiens2 and Bremen (data not shown). In analysis of only the Kashmir populations ΔK suggested 3 (ΔK = 324.75, H = 0.949) as the number of K best describing the data, while the CLUMPP H value was the highest for K = 2 clusters (ΔK = 11.375, H = 0.972, Additional file 4). The K = 2 cluster model primarily separated Kashmir2 from Kashmir1 and Kashmir3, while at K = 3 all populations consisted of individuals assigned to different clusters (data not shown).

We additionally evaluated our data for genetic structuring using discriminant analysis of principal components (DAPC), which is free of the assumptions of HWE and no LD present in STRUCTURE. The number of DAPC clusters best describing the different data sets was not clear-cut for the full and European data sets (Additional file 5), but the automatic selection implemented in *find.clusters* suggested similar or higher numbers of clusters to those found by the STRUCTURE analysis (all data K = 2, European data K = 5, Kashmir data K = 2). As our primary aim was to evaluate how the violation of STRUCTURE assumptions affected the clustering we compared the results from the STRUCTURE analyses with the highest support to those from the DAPC analyses with the same number of clusters. The DAPC results showed a high degree of correspondence with the outcome of the STRUCTURE analyses suggesting that the effect of LD and deviation from HWE on the analyses had been minor.

### Support of independent colonisations from approximate Bayesian computation but not principal component analyses

**Fig. 3:**
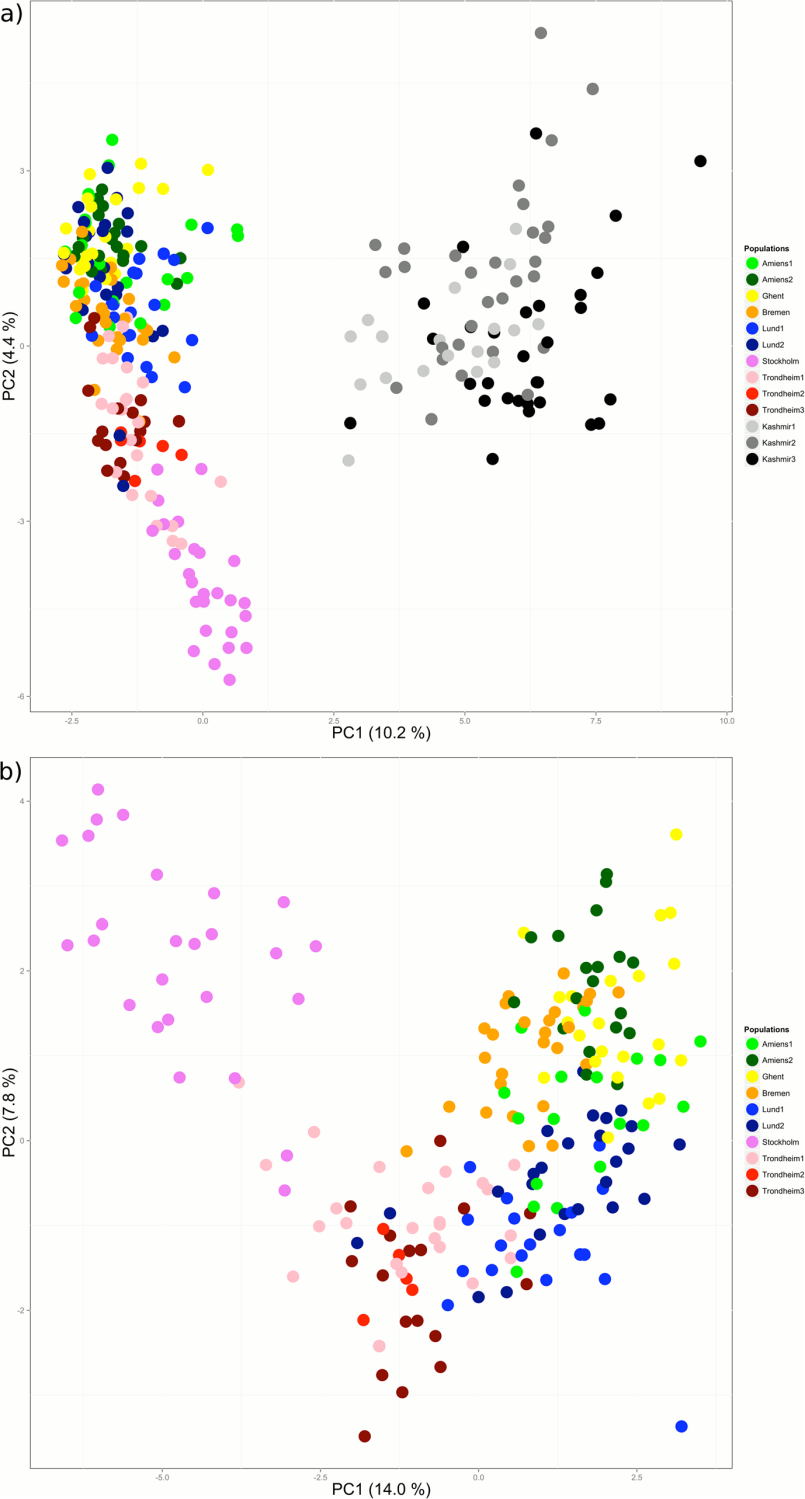
PCA for **a**) all populations and **b**) all sampled European populations with outlier genotypes for Amiens1 individuals removed

Principal component analysis (PCA) of the full dataset clearly separated the Kashmir (black and grey) from the European populations (in colour) (Fig. 3a) along the first two principal components (PCs). The wider spread of Kashmir individuals along PC1 and PC2 (Fig. 3a) reflected the higher genetic diversity present in the Kashmir populations. Analysis of only the European individuals showed three individuals from Amiens1 to be highly divergent (data not shown). This proved to be the result of their genotypes at the A2 locus and excluding these genotypes from the analysis mostly removed the divergence of these individuals. After removal of the deviant A2 genotypes almost all the individuals of the Stockholm population clustered separately from all other European individuals, while the rest showed partial overlap with a gradual transition across a roughly geographical gradient (Fig. 3b) (correlation latitude vs PC1: r = −0.653; latitude vs PC2: r = −0.576; longitude vs PC1: r = −0.710; longitude vs PC2: r = −0.184; all *p* < 0.001). The north – south clustering found in the STRUCTURE analysis was not apparent in the PCA (Fig. 3b).

We postulated that the two regional clusters detected in the STRUCTURE and DAPC analyses, southern and northern Europe, could be the result of independent introductions into Europe. In our approximate Bayesian computation (ABC) modelling, posterior probability values (Table 2) consistently supported a scenario where the separation of the European regional clusters occurred after their separation from the Kashmir populations (scenario 1 in Additional file 6), although with a type II error of 0.152. This order of separation is expected in a scenario with a single colonisation event. However, the median values of the time since the separation of the different clusters were 992 (separation of European clusters, q_0.05_ = 292, q_0.95_ = 3220) and 4850 generations (separation of Kashmir cluster, q_0.05_ = 1670, q_0.95_ = 9260) respectively. Similar estimates of separation time were obtained when only the European populations were analysed, where the time back to separation of the southern and northern European regions had a median value of 342 generations (q_0.05_ = 77.5, q_0.95_ = 2310). In both cases ABC modelling supported a separation of the two European regions predating their introduction in Europe during the 19^th^ and 20^th^ centuries although a postintroduction separation was not fully excluded by the analysis of European populations only.

**Table 2:** Posterior probabilities with 95 % confidence intervals (in brackets) for the two scenarios used in ABC analysis of the population history of the full Impatiens glandulifera dataset. Posterior probabilities were measured using the 50 and 1000 closest datasets for the direct and logistic approaches respectively, out of 1 000 000 simulated datasets. Model scenarios as presented in Additional file 6

	Posterior probabilities	
Scenario	Direct approach	Logistic approach
1	100 %	99.98 % (99.72 – 100)
2	0 %	

### *Genetic differentiation between* I. glandulifera *populations*

Analysis of molecular variance (AMOVA) showed significant genetic structure among the 13 populations and higher hierarchical levels (Table 3). As expected the differentiation was higher between continents, Kashmir and Europe, than among populations within continents, but also higher among the seven municipalities than among populations within municipalities (Table 3). This suggests either limitations to gene flow, high genetic drift or the remnants of earlier founder effects not only between Kashmir and Europe, but also among different municipalities. Analysing the European data only showed that differentiation was lower among populations within municipalities than among municipalities (Table 4). Significant differentiation was found between northern and southern Europe, but differentiation among populations within regions was higher than between regions (Table 4). Looking at Kashmir only, differentiation at the population level was somewhat lower (Table 5), indicative of a less restricted gene flow in the native range, although the difference between Kashmir and Europe could also be the result of the European populations not yet having reached drift – migration equilibrium.

**Table 3:** Results from AMOVA of all sampled *Impatiens glandulifera* populations

	Continents			Municipalities		
	% variance explained	F-statistic	*p*	% variance explained	F-statistic	*p*
Between continents / among municipalities (F_CT_)	35.22	0.352	<0.01	29.25	0.292	<0.001
Among populations^a^ (F_SC_)	16.81	0.260	<0.001	10.58	0.150	<0.001
Within populations (among and within individuals) (F_IS_)	47.96	−0.038	>0.05	60.17	0.187	>0.05

Percentage of variance of genetic diversity explained between continents or among municipalities, among populations and within populations, F-statistics and p values for the different hierarchical levels. The dataset was analysed both with continent as the highest hierarchical level (second through fourth columns) and with municipality as the highest hierarchical level (fifth through seventh columns)

^a^Percentage variation among populations within continents (first column) and municipalities (third column)

**Table 4:** Results from AMOVA of European *Impatiens glandulifera* populations

	Regions			Municipalities		
	% variance explained	F-statistic	*p*	% variance explained	F-statistic	*p*
Between regions / among municipalities (F_CT_)	14.15	0.145	<0.05	21.76	0.218	<0.05
Among populations^a^ (F_SC_)	23.78	0.277	<0.001	13.38	0.171	<0.001
Within populations (among and within individuals) (F_IS_)	62.07	−0.136	>0.05	64.86	−0.136	>0.05

Data as in Table 3. The dataset was analysed both with region as the highest hierarchical level (second through fourth columns) and with municipality as the highest hierarchical level (fifth through seventh columns)

^a^Percentage variation among populations within regions (first column) and municipalities (third columns)

**Table 5:** Results from AMOVA of Kashmir *Impatiens glandulifera* populations

	% Variance explained	F-statistic	*p*
Among populations (F_ST_)	11.39	0.114	<0.001
Within populations (among and within individuals) (F_IS_)	88.60	0.085	<0.05

Data as in Table 3

Pairwise F_ST_ values (Table 6) between all possible pairs of one southern European and one northern European population were of a similar magnitude as F_ST_ values between all possible pairs of one European and one Kashmir population (Wilcoxon rank sum test, *p* = 0.083). F_ST_ values were lower between pairs of Kashmir populations (mean 0.102, s.d. 0.026) than between pairs of European populations (mean 0.414, s.d. 0.165, Wilcoxon rank sum test *p* < 0.001), and the difference was not driven by the larger distances covered in the European sampling. This was shown by the fact that F_ST_ values only for within-municipality pairs of populations (mean 0.243, s.d. 0.113) were also significantly higher than F_ST_ values for the Kashmir populations (Wilcoxon rank sum test *p* < 0.05).

**Table 6:** Pairwise F_ST_ values for all pairs of populations of *Impatiens glandulifera*

	Amiens2	Ghent	Bremen	Lund1	Lund2	Stockholm	Trondheim1	Trondheim2	Trondheim3	Kashmir1	Kashmir2	Kashmir3
Amiens1	0.431	0.130	0.192	0.191	0.063	0.558	0.369	0.590	0.472	0.443	0.467	0.510
Amiens2		0.404	0.338	0.562	0.401	0.638	0.513	0.728	0.651	0.502	0.530	0.549
Ghent			0.269	0.379	0.181	0.598	0.454	0.626	0.547	0.465	0.493	0.530
Bremen				0.307	0.196	0.487	0.368	0.540	0.431	0.403	0.453	0.485
Lund1					0.231	0.572	0.385	0.561	0.455	0.481	0.480	0.528
Lund2						0.556	0.336	0.605	0.447	0.460	0.486	0.524
Stockholm							0.381	0.470	0.496	0.348	0.390	0.421
Trondheim1								0.159	0.188	0.371	0.436	0.459
Trondheim2									0.164	0.531	0.557	0.579
Trondheim3										0.468	0.505	0.534
Kashmir1											0.093	0.082
Kashmir2												0.131

### *Isolation by distance in European* I. glandulifera

We evaluated isolation by distance within Europe using four different measures of genetic differentiation between pairs of populations: pairwise genetic distance, proportion of shared alleles and pairwise F_ST_ and R_ST_ values in the form of F_ST_/(1-F_ST_) and R_ST_/(1-R_ST_) respectively (Table 6, Additional file 7). Geographic distance was related to F_ST_/(1-F_ST_) and genetic distance (Mantel test, F_ST_/[1-F_ST_]: *p* < 0.001, r^2^ = 0.372; D_CH_: *p* < 0.01, r^2^ = 0.295) but not to the proportion of shared alleles or R_ST_/(1-R_ST_) (Mantel test, proportion of shared alleles: *p* = 0.999, r^2^ = 0.030; R_ST_/[1-R_ST_]: *p* = 0.08, r^2^ = 0.204).

Looking at the regional clusters detected by STRUCTURE, the northern populations showed signs of isolation by distance when genetic similarity between populations was measured as F_ST_/(1-F_ST_) or R_ST_/(1-R_ST_) (Mantel test both instances *p* < 0.05, r^2^ = 0.889 and 0.587 respectively), while the southern populations showed signs of isolation by distance when genetic similarity between populations was measured as R_ST_/(1-R_ST_) (Mantel test R_ST_/[1-R_ST_]: *p* < 0.01, r^2^ = 0.185; for all other comparisons in the northern and southern region *p* > 0.05). The presence of isolation by distance among the northern populations was mainly created by the large genetic distances between the single Stockholm population and all three Trondheim populations and did not persist when Stockholm was removed (Mantel test, all *p* > 0.05).

### *Limited effects of mutation, migration and bottlenecks in European* I. glandulifera

Pairwise R_ST_ values did in most cases not differ from the F_ST_ values (Table 6, Additional file 7) (Amiens1 vs Kashmir2, Amiens2 vs Kashmir2, Amiens2 vs Kashmir3, Ghent vs Bremen and Kashmir1 vs Kashmir2: *p* < 0.05; Kashmir2 vs Kashmir3 *p* < 0.01; all other comparisons *p* > 0.05) suggesting that mutation has been of limited importance in differentiating populations both within and between continents. A limited role of mutation in Europe was further supported by the fact that only four private alleles were found in the ten European populations, compared to the 20 private alleles that were found in the three Kashmir populations studied.

Populations that have recently undergone a bottleneck will experience both loss in the number of alleles and observed heterozygosity. In spite of the relatively recent naturalisation and spread across Europe we found little evidence of genetic bottlenecks when analysing the data with the software BOTTLENECK. No heterozygosity excess was detected for any population in any of the three population group sets used (Wilcoxon sign-rank text, all *p* > 0.05). However, two of the northernmost populations, Trondheim1 and Trondheim2, showed the shifted mode indicative of a recent bottleneck. The proportion of migrants into a population, assessed using the software BayesAss, was in most cases less than 1 %, and only a few populations showed indications of more than 10 % of the individuals being migrants from other populations (Additional file 7). The highest migration rates were shown within municipalities, from Trondheim2 to Trondheim1, and from Lund1 to Lund2, but also from Lund1 to Amiens1 and Bremen. The migrant individuals suggested were in all cases 1^st^ generation migrants.

### Genetic trends and patterns in invasive plants

Comparing the genetic diversity of invasive plant species in their native and invasive ranges from 39 published studies showed that genetic diversity in the native ranges was significantly higher than the diversity in the invasive ranges (Additional file 8, paired Wilcoxon rank sum test, *p* < 0.01). A diversity in the invasive range similar to that of the native range was, however, not uncommon. In the 41 comparisons that identified a number of introductions, the majority, 32, suggested multiple introductions and only five a single origin of the invasive species (Additional file 8).

The species reviewed did not have significantly higher F_ST_ values in the invasive compared to the native ranges (Additional file 8, paired Wilcoxon rank sum test, *p* = 0.052). Although small population sizes in newly introduced species could lead to an increase in the amount of inbreeding in a species, there was no significant difference in the inbreeding coefficients of the native and invasive ranges of species reported in the literature to be outbreeding (Additional file 8, paired Wilcoxon rank sum test, *p* = 0.651).

The distribution of genetic diversity within and among populations, as analysed by AMOVAs, showed that within each species similar amounts of variation were present within and among populations in the native and invasive ranges (Additional file 8). The AMOVAs also showed that the distribution of genetic diversity differed drastically from species to species (Additional file 8).

## Discussion

### *Source population of European* I. glandulifera

The present study lends support to the notion that high genetic diversity is not a prerequisite for becoming a thriving invasive species. *I. glandulifera* thus adds to the list of successful invaders shown to have limited genetic diversity in their invasive compared to native ranges [2, 22, 27, 50, 76, 84, 108]. Most of the invasive species with a low genetic diversity in their invasive range are, however, species that reproduce apomictically or autogamously. Among the studies reviewed *Acacia saligna* [50] was the only outcrosser to have a genetic diversity that was lower in its invasive compared to native range than the equally outcrossing *I. glandulifera* studied by us. The low genetic diversity of *I. glandulifera* is also remarkable in the light of possible repeated introductions.

The confident identification of the true source population(s) of any invasive species typically requires a wider and denser sampling of the native range than the one in the present study. Our ABC modelling suggests that the Kashmir populations sampled in this study are not the direct source of the European populations studied. The separation time between the Kashmir *I. glandulifera* and either European cluster is at least several hundred years, indicating that the source population(s) of the European *I. glandulifera* most likely separated from the Kashmir populations at least a couple of hundred years before the species was introduced in Europe [57]. Indications of ascertainment bias from the higher presence of null alleles in Kashmir populations further suggest that it is not the source of European *I. glandulifera*. A wider sampling of *I. glandulifera*, preferably from its full native range, will be needed if possible sources for the populations in Europe are to be identified.

The fact that the Kashmir populations in this study are not the source population of *I. glandulifera* in Europe limits our ability to make inferences about colonisation processes, such as the exact amount of loss of genetic diversity during colonisation. We note, however, that all but four of the alleles detected in the European populations were also present in the Kashmir populations suggesting that the alleles present in Kashmir populations to a large extent represent those of the source of the European populations. While we sampled a large number of individuals from few populations in the species native range, Nagy and Korpelainen [64] sampled mostly four or fewer individuals from a larger area covering both India and Pakistan. The two sampling regimes showed a similar number of alleles (using an overlapping set of markers) and similar amounts of within-population genetic diversity for India and Pakistan [64] and Kashmir (this study) suggesting that the Kashmir populations studied here well represent the average levels of genetic diversity of a significant part of the species’ native range. Additionally, the STRUCTURE analysis performed by Nagy and Korpelainen [64] suggested that the nine populations sampled by them in India and Pakistan all belonged to the same cluster. Since our populations lie within the area studied by Nagy and Korpelainen [64] it is likely our populations would have fallen within the same cluster, though the levels of genetic diversity need not to be comparable. Nepalese populations of *I. glandulifera* have yet to be studied genetically, but our Kashmir populations most likely sufficiently well characterize populations in the species’ native range for us to draw tentative conclusions regarding the genetic differences between *I. glandulifera* in its native and introduced ranges.

### *Introduction history of* I. glandulifera

The presence of *I. glandulifera* in Europe was reported from gradually more northern locations (see e.g. [37, 47, 67, 73, 97]), suggesting a progressive northward spread of the species during the early 20^th^ century. In such a scenario latitudinal effects on different measures of the distribution of genetic diversity could be expected as the result of successive colonisation events. However, multiple introductions seem to be the norm for invasive species (Additional file 8) and repeated introductions have been proposed for *I. glandulifera* in Finland [64]. We found little evidence of latitudinal effects on the different measures of genetic diversity and equally strong correlations between the distribution of genetic diversity (Fig. 3b) and latitude as longitude. A possible explanation could be that isolation by distance rather than gradual northward colonisation is responsible for the pattern observed in our PCA. However, our comparisons of geographical and genetic distances showed limited support for isolation by distance and implied that at least the traces of isolation by distance detected in the north are driven by the Stockholm population.

Although our PCA showed a gradual transition from more southern to more northern populations (Fig. 3b) this was not supported by the STRUCTURE analyses (Fig. 2). Instead, STRUCTURE separated the European gene pool into a northern regional cluster, consisting of central Sweden and Norway, and a southern regional cluster, with all remaining European populations, with no gradual transition in cluster identity among the populations studied. The discrepancy between the STRUCTURE analysis and the PCA could be the result of limitations in handling patterns of isolation by distance by STRUCURE [62] or by differences in how missing data was handled in the two methods. The presence of isolation by distance has also been shown to bias tests of AMOVA [62] and our AMOVA support of a regional division should thus also be interpreted with caution. It is also worth noting that the AMOVA of European data found more differentiation within the regions detected by STRUCTURE than between regions thus supporting the PCA results rather than those from the STRUCTURE analysis.

A stronger support for the regional division comes from our ABC modelling of the population history of the samples where estimates of the time of separation for southern European and northern European *I. glandulifera* show that it most likely predates the species introduction in Europe. If the regional division is an artefact of isolation by distance we expect populations in the two regions to have separated from each other only after the species colonised Europe. Although the ABC modelling produced a large range for the estimates of the time since separation and a divergence after the introduction in Europe is possible from the analysis of European data only, a separation pre-dating the introduction in Europe is more likely and suggests at least two independent introductions. The fact that Stockholm individuals cluster away from all other European individuals in the PCA (Fig. 3b) and in the four-cluster STRUCTURE analysis of European individuals tentatively suggests this might be the result of yet another introduction. In conclusion, we find support for multiple introductions of *I. glandulifera* but note the possibility of it also being an artefact of the presence of isolation by distance.

Although there are records of seeds and seedlings being brought to Europe from Russia and India in addition to the first introduction to the Kew Gardens [47], it is not clear from which introductions present day European plants of *I. glandulifera* descend. In addition, it is not clear whether the Finnish populations studied by Nagy and Korpelainen [64] belong to the Northern European cluster detected in this study. Comparisons of the populations studied here with British and Finnish populations will be needed to elucidate the relationship between the populations in this study, the original introduction to Kew Gardens and the multiple introductions suggested by Nagy and Korpelainen [64].

### Genetic diversity after the colonisation of invasive ranges

A number of studies comparing the genetic diversity of invasive plants in their native and introduced ranges have been carried out in a range of different species (Additional file 8). The different studies have used contrasting types of genetic markers and different approaches to sample the species in their native and introduced ranges. More studies will be needed in order to test the effects of factors such as growth habit, mode of reproduction and life span of the species on the population genetics of plant invasion. In spite of this some general trends can be discerned and tentative conclusions can be drawn from the studies available in the literature.

A general loss in genetic diversity upon invasion is apparent in the studies reviewed by us (Additional file 8) and has also been noted in plants previously [102]. Although we have not sampled the source population of European *I. glandulifera*, and our results should be interpreted with caution, it is likely that the Kashmir populations are representative enough that conclusions can still be drawn. The low total genetic diversity after introduction in *I. glandulifera* is a more drastic reduction (55 %) than that detected in many invasive species so far, with an average diversity reduction of 11 % (Additional file 8). Even when restricting our comparison to species with only a single or a few reported introductions the proportion of post- to pre-introduction genetic diversity was lower in *I. glandulifera* than in most other species (average increase of 2 %, Additional file 8). This may at least in part be explained by the high inter-annual variation in population size in *I. glandulifera*, where large populations can decline dramatically in size and sometimes go extinct [30]. This leads to increased genetic drift and potentially higher loss of genetic diversity than in species with more stable population sizes.

The reduction in diversity detected was, however, also larger than the one reported by Nagy and Korpelainen [64] for four Finnish populations (48 %) and a UK population (26 %). Different introductions giving rise to the populations in these studies could contribute different amounts of genetic diversity, through the introduction of different number of individuals or individuals carrying different amounts of diversity. Additionally, post-introduction genetic drift can differ between populations. Although the even distribution of genetic diversity over Europe and the generally low number of private alleles suggest a primary role of founder effects, the fact that between any two European populations only about 32 % of the alleles are shared (34 % and 36 % in the southern and northern European clusters, respectively), indicates that also post-introduction genetic drift, introductions from other parts of the native range or other evolutionary processes have played a role. Like invasive species in general (Additional file 8), invasion of *I. glandulifera* in Europe, however, seems not to have been accompanied by an increase in the level of inbreeding.

Bottlenecks, such as those occurring during the colonisation of a new area, are expected to lead to a loss of genetic diversity [66, 69, 102] and diversity levels can change during the course of a species invasion history [101]. Genetic diversity can, for example, be lost through high genetic drift in small populations colonizing new parts of the invasive range. Only among the northernmost populations could we detect the effects of past population bottlenecks. It has, however, been pointed out that even strong bottlenecks can be difficult to detect with heterozygosity-excess based tests such as the one implemented in BOTTLENECK [74], and undetected bottlenecks could have occurred in the recent history of additional populations. Furthermore, if the sampled populations correspond to biological populations, the pooling of populations in the two- and three-population group sets (as suggested from STRUCTURE analysis) may have inflated the amount of homozygosity and reduced our capacity of detecting excess heterozygosity. Of the populations indicated as having undergone a bottleneck from the shifted allele frequency distribution, the population Trondheim2, was particularly conspicuous with few alleles and a low genetic diversity. In addition the highly negative inbreeding coefficient from excess heterozygosity could be a consequence of among other things a recent bottleneck, disassortative mating or higher than random rates of outcrossing in this population.

Beyond the populations Trondheim1 and Trondheim2 we found little direct evidence of past bottlenecks, though European populations of *I. glandulifera* had still, on average lost as much as about 70 % of the diversity of the populations in the species’ native range in Kashmir. This was true for both number of alleles and genetic diversity, suggesting major loss of genetic diversity during the introduction to Europe and subsequent spread of the species. The loss of diversity is particularly striking given the small geographical area sampled in Kashmir.

### *The causes of genetic differentiation between* I. glandulifera *populations*

In a newly introduced plant species, genetic differentiation between populations can be expected to be low if mutation, selection and genetic drift have not yet led to diversification of populations. Such lack of genetic structuring has, for example, been found in the invasive range of *Macfadyena unguis-cati* [76] and *Olea europea* [7], both believed to have a single introduction into its invasive range [76]. At the same time repeated founder events, or introductions from different sources, could cause populations to become genetically differentiated from each other (e.g. [14, 64, 84, 89]).

From our F_ST_ – R_ST_ comparisons, mutation seems not to have played a major role in the differentiation of European populations during the evolutionary short time since their introduction, nor in the differentiation of the Kashmir populations. Of the six cases where F_ST_ differed significantly from R_ST_ four involved the population Kashmir2. Interestingly, we found significant differences also between Kashmir2 and the two other Kashmir populations, located only a few kilometers apart, even though it seems unlikely that these populations have been separated long enough for mutation to cause significant amounts of differentiation.

Our AMOVA suggested fairly high differentiation among municipalities and to a lesser extent among populations. This could either be the result of limited gene flow, a founder effect or both, though the bottleneck usually associated with a founder event was not strongly supported in our data. F statistics (assuming migration – drift equilibrium) suggested that gene flow may be higher between the Kashmir populations than the European ones. Although higher F_ST_ values in the invasive range is consistent with some invasive species, invasive plants in general were found not to differ in F_ST_ values or the distribution of genetic diversity as shown by AMOVAs (Additional file 8). Our findings concerning *I. glandulifera*, however, contrasted the distribution of genetic variance previously found in Lithuanian plants where a much higher percentage of the genetic diversity was found among populations [110]. It should be noted, however, that the study by Zybartaite et al. [110] was based on RAPD markers and that the reliability of this type of markers has been questioned [63].

We see our estimates of migration rates primarily as an indication of populations more likely to have experienced gene flow. As could have been expected, most of the migrants detected were from neighbouring populations (from Trondheim2 to Trondheim1, 1.8 km, and from Lund1 to Lund2, 2.3 km). The longer migrations detected (600 km or more), if not a consequence of few markers and low diversity, should be the result of man-mediated dispersal. The latter seems a likely scenario between Lund1 and Amiens1 where as many as eight individuals were identified as possible first generation migrants. High within-municipality F_ST_ values from Lund and Amiens were partially the reason why within-municipality F_ST_ values were higher in Europe than in Kashmir and it is possible that man-mediated dispersal to single populations in these municipalities has contributed to the high F_ST_ values observed. The ornamental qualities ascribed to *I. glandulifera* [1], and the presence of *I. glandulifera* in such isolated locations in Britain such as the Isles of Scilly, Shetland and Orkney [6] point to the importance of anthropogenic spread of the species. This study further supports the role of man, in particular when it comes to repeated introductions and long distance spread, in playing an important role in shaping the distribution of genetic diversity in *I. glandulifera*.

## Conclusions

In conclusion, we find that invasive populations of *I. glandulifera* represent a smaller proportion of the genetic diversity of the native range than what is typically found for outcrossing invasive species. Relatively low genetic diversity is thus possible in widespread invasive species even after multiple introductions. This suggests a possible role for phenotypic plasticity in facilitating the spread of *I. glandulifera* across Europe.

## Methods

### Plant material

Populations of *I. glandulifera* (2n = 18, 20) with at least 30 flowering individuals were sampled in 2011 from six municipalities, the local area around a town or city, along a 1600 km latitudinal gradient in Western Europe. Populations were also sampled from Kashmir, India (Fig. 1), the part of the native range suggested to be the source of the plants originally introduced into Europe [6, 57] (Table 1). The populations ranged in size from less than 100 individuals to more than 1000 and were located both nearby and away from waterways. In each municipality, one to three populations were sampled (Table 1) with a minimum distance of 1.8 km between each population. The maximum distance between the Kashmir populations was 6.4 km. From each population leaf material from 30 randomly sampled individuals was collected and stored in tubes containing silica gel until used for analysis. Sampling was done according to national legislations and samples were destroyed upon molecular analysis. A map showing the location of the sampled populations (Fig. 1) was drawn using Ocean Data View version 4.7.2 [88].

### Molecular analysis

Plant tissue was dried overnight at 45 °C, after which total DNA was extracted using the E-Z 96 Plant DNA Kit (Omega Bio-tek Inc., Norcross, GA, USA), following the manufacturer’s recommendations. Eleven already published microsatellite loci [78, 100] were used for PCR amplification. Multiplex PCR was performed with the Qiagen® Multiplex PCR Kit (Qiagen) using a 17.5 μl reaction with 1 x Qiagen Mastermix and 0.1 – 0.4 M of each primer. PCR was carried out with an initial 15 min denaturation at 95 °C followed by 30 cycles of 94 °C for 30 s, 55 °C for 90 s and 72 °C for 60 s with a final elongation for 10 min at 72 °C. Microsatellite lengths were determined by running the PCR products on a Genetic Analyzer 3130xl (Applied Biosystems, CA, USA), and the resulting data were analysed with Geneious v 6.1.8 (BiomatesLtd, Auckland, New Zealand).

### Data analysis

The expected heterozygosity under HWE (h) was calculated with a purpose-written Perl script (available upon request). GenePop [82, 86] was used to test for deviations from HWE. FreeNA [13] was used to test for the presence of null alleles and for obtaining estimates of F_ST_ [105] and D_CH_, the Cavalli-Sforza and Edwards genetic distance [12]. Adjusting genetic diversity, F_ST_ and D_CH_ for the presence of null alleles did not affect the conclusions drawn and these measures are therefore not reported. D_S_ (Nei’s genetic distance [65]) and R_ST_, an equivalent of F_ST_ using a stepwise mutation model, [85, 91] were calculated using Spagedi v 1.4 [35] and differences between R_ST_ and F_ST_ were tested using the permutation test implemented in the software. D_CH_ and D_S_ were highly correlated (r = 0.949, Pearson’s product–moment correlation, p < < 0.001) and only the former is reported.

Hierarchical AMOVA, F_IS_ and tests for LD (using 10000 permutations) were calculated and carried out using Arlequin v 3.5.1.3 [25]. For AMOVA of the full dataset continent and municipality were both used as the highest hierarchical level while the European dataset was analysed with both region and municipality as the highest hierarchical level. Analyses with municipality as the highest hierarchical level included also municipalities for which only a single population was available. Bayes-Ass v 3.0 [107] was used to estimate migration between populations. BOTTLENECK v 1.2.02 [16] was used to detect recent population bottlenecks among the studied populations. For the BOTTLENECK analysis, a two-population group set (all European individuals and all Kashmir individuals, respectively), a three-population group set (northern European individuals from Stockholm and Trondheim, remaining southern European individuals and Kashmir individuals respectively) and a 13-population group set (each sampled population treated as a separate unit) were analysed under a stepwise mutation model. Due to the low number of markers analysed heterozygosity was tested using the Wilcoxon sign-rank test.

We examined geographic clustering of the genetic diversity using a combination of different approaches. STRUCTURE v 2.2 [26, 77] was run with a burn-in length of 20 000 iterations followed by 50 000 iterations for estimating the parameters, with non-amplifying markers treated as missing data. Each analysis was repeated ten times for each number of clusters (K = 1 to 20) until the likelihood values for the runs no longer improved. We evaluated both the admixture and the no admixture models, but as the two models gave similar results we only report the former. The number of clusters observed in the dataset was evaluated by calculating ΔK according to Evanno et al. [24]. CLUMPP v 1.1.1 [42] was used to compare the results of individual runs and to calculate similarity coefficients, H, and the average matrix of ancestry. In CLUMPP, the FullSearch, Greedy and LargeKGreedy algorithms were used for comparing runs with K < 4, K 4 – 6 and K > 6, respectively. Graphical presentation of the results was obtained using DISTRUCT v 1.1 [83].

Geographic structure was further explored using R v 3.0.3 [81] to carry out DAPC analysis [43], using the *Adegenet* package, and PCA. The number of copies present in an individual (0, 1 or 2) for each allele at each locus was treated as independent variables in the PCA. In addition, R was used to test for correlations between latitude and genetic diversity and the two main principal components in PCA respectively as well as between geographic distance and pairwise F_ST_, R_ST_, number of alleles and genetic distance respectively, using Pearson’s product–moment correlation. R was also used to carry out t-tests, or alternatively the Wilcoxon rank sum test if the underlying assumptions of parametric tests were not met.

We used the ABC approach implemented in DIYABC [17] to infer past demographic history and try to distinguish between one or two origins of *I. glandulifera* in Europe. We compared two models, one where all European populations first separate from the Kashmir populations followed by a later split into a southern and a northern regional cluster, and one with a southern regional cluster first splitting from the remaining populations, followed by a second split between a northern cluster and the Kashmir populations (Additional file 6). In addition only European populations were analysed in a scenario where a single ancestral population split into the southern and northern regional clusters. Both the direct and the logistic approaches implemented in DIYABC were used and parameter priors for the different scenarios including effective population sizes, time of splitting and mutation models are given in Additional file 9.

### Literature study

In addition to literature reviewed in Bossdorf et al. [8] and Dlugosch and Parker [21], Google Scholar was in May 2014 queried using “plant” together with “invasive”, “alien” or “exotic”. From the resulting studies from all publication years those reporting genetic diversity measures of invasive species both in their native and invasive ranges were chosen and the diversity of the two ranges was compared.

### Availability of supporting data

The data set supporting the results of this article is available from the Dryad repository http://dx.doi.org/10.5061/dryad.gp2tc [34].

## Additional files

Additional file 1:
**Description of the loci studied, divided by population.** Frequency of null alleles as estimated by the Expectetion Maximization algorithm implemented in FreeNA, and loci with allele frequencies significantly deviating from Hardy Weinberg equilibrium (HWE). Additional file 2:
**Populations showing significant linkage disequilibrium (LD) between pairs of loci.**
Additional file 3:
**Genetic diversity (h) of** ***I. glandulifera*** **for the different markers studied for observed allele frequencies.**
Additional file 4:
**Likelihood values, ΔK values and CLUMPP H values for the different STRUCTURE runs.**
Additional file 5:
**BIC values for DAPC analyses of full, European and Kashmir data sets respectively.**
Additional file 6:
**Illustration of scenarios used in ABC analyses of population history with the recent history at the bottom of the panels and the past history at the top.** Pop 1 = Southern European individuals, pop 2 = Northern European individuals, pop 3 = Kashmir individuals, with different colours denoting differences in population size. Scenario 1: Initial separation of all European individuals from Kashmir individuals at t2 followed by separation of the two European populations from each other at t1. Scenario 2: Initial separation of southern European individuals from northern European and Kashmir individuals at t2 followed by a later separation of the northern European individuals from the Kashmir ones at t1. Additional file 7:
**Pairwise comparisons of all pairs of populations of** ***Impatiens glandulifera.*** Pairwise genetic differentiation measured as R_ST_, proportion of shared alleles and D_CH_, and proportion migrants between pairs of populations as estimated by BayesAss. Additional file 8:
**Studies comparing genetic diversity in native and introduced ranges of invasive species.**
Additional file 9:
**Prior distribution of parameters used in ABC analysis.**

## Abbreviations

HWE: Hardy-Weinberg Equilibrium
LD: Linkage disequilibrium
DAPC: Discriminant analysis of principal components
PCA: Principal component analysis
PC: Principal component
ABC: Approximate Bayesian computation
AMOVA: Analysis of molecular variance

## References

[1] Anon (1843). *Impatiens glandulifera*. Glandular Balsam; Touch me not. Curtis’s Botanical Magazine.

[2] Bakker EG, Montgomery B, Nguyen T, Eide K, Chang J, Mockler TC (2009). Strong population structure characterizes weediness gene evolution in the invasive grass species *Brachypodium distachyon*. Mol Ecol.

[3] Balfourier F, Charmet G (1994). Geographical patterns of isozyme variation in Mediterranean populations of perennial ryegrass. Heredity.

[4] Barrett SC, Husband BC, Brown A, Clegg M, Kahler A, Weir B, Brown AHD, Clegg MT, Kahler AL, Weir BS (1990). The genetics of plant migration and colonization. Plant population genetics, breeding, and genetic resources.

[5] Beerling DJ (1993). The Impact of Temperature on the Northern Distribution-Limits of the Introduced Species *Fallopia japonica* and *Impatiens glandulifera* in north-west Europe. J Biogeogr.

[6] Beerling DJ, Perrins JM (1993). Biological flora of the British Isles: *Impatiens glandulifera* Royle (*Impatiens roylei* Walp.). J Ecol.

[7] Besnard G, Henry P, Wille L, Cooke D, Chapuis E (2007). On the origin of the invasive olives (*Olea europaea* L., Oleaceae). Heredity.

[8] Bossdorf O, Auge H, Lafuma L, Rogers WE, Siemann E, Prati D (2005). Phenotypic and genetic differentiation between native and introduced plant populations. Oecologia.

[9] Britten J. *Impatiens roylei* in England. J Bot. 1900;38.

[10] Burdon JJ, Brown AHD (1986). Population genetics of *Echium plantagineum* L. – Target weed for biological control. Aust J Biol Sci.

[11] Buswell JM, Moles AT, Hartley S (2011). Is rapid evolution common in introduced plant species?. J Ecol.

[12] Cavalli-Sforza LL, Edwards AW (1967). Phylogenetic analysis. Models and estimation procedures. Am J Hum Genet.

[13] Chapuis MP, Estoup A (2007). Microsatellite null alleles and estimation of population differentiation. Mol Biol Evol.

[14] Chun YJ, Nason JD, Moloney KA (2009). Comparison of quantitative and molecular genetic variation of native vs. invasive populations of purple loosestrife (*Lythrum salicaria* L., Lythraceae). Mol Ecol.

[15] Coombe DE (1956). Notes on some British plants seen in Austria. Veröffentlichungen des Geobotanischen Instituts, Eidgenössiche Technische Hochschule Rübel in Zürich.

[16] Cornuet JM, Luikart G (1996). Description and power analysis of two tests for detecting recent population bottlenecks from allele frequency data. Genetics.

[17] Cornuet JM, Pudlo P, Veyssier J, Dehne-Garcia A, Gautier M, Leblois R (2014). DIYABC v2.0: a software to make approximate Bayesian computation inferences about population history using single nucleotide polymorphism, DNA sequence and microsatellite data. Bioinformatics.

[18] Dawson FH, Holland D (1999). The distribution in bankside habitats of three alien invasive plants in the UK in relation to the development of control strategies. Hydrobiologia.

[19] DeWalt SJ, Hamrick JL (2004). Genetic variation of introduced Hawaiian and native Costa Rican populations of an invasive tropical shrub, *Clidemia hirta* (Melastomataceae). Am J Bot.

[20] DeWalt SJ, Siemann E, Rogers WE (2011). Geographic distribution of genetic variation among native and introduced populations of Chinese tallow tree, *Triadica sebifera* (Euphorbiaceae). Am J Bot.

[21] Dlugosch KM, Parker IM (2008). Founding events in species invasions: genetic variation, adaptive evolution, and the role of multiple introductions. Mol Ecol.

[22] Dlugosch KM, Parker IM (2008). Invading populations of an ornamental shrub show rapid life history evolution despite genetic bottlenecks. Ecol Lett.

[23] Durka W, Bossdorf O, Prati D, Auge H (2005). Molecular evidence for multiple introductions of garlic mustard (*Alliaria petiolata*, Brassicaceae) to North America. Mol Ecol.

[24] Evanno G, Regnaut S, Goudet J (2005). Detecting the number of clusters of individuals using the software STRUCTURE: a simulation study. Mol Ecol.

[25] Excoffier L, Lischer HE (2010). Arlequin suite ver 3.5: a new series of programs to perform population genetics analyses under Linux and Windows. Mol Ecol Resour.

[26] Falush D, Stephens M, Pritchard JK (2003). Inference of population structure using multilocus genotype data: Linked loci and correlated allele frequencies. Genetics.

[27] Fennell M, Gallagher T, Osborne B (2010). Patterns of genetic variation in invasive populations of *Gunnera tinctoria*: an analysis at three spatial scales. Biol Invasions.

[28] Frankham R (2005). Resolving the genetic paradox in invasive species. Heredity.

[29] Gaudeul M, Giraud T, Kiss L, Shykoff JA (2011). Nuclear and chloroplast microsatellites show multiple introductions in the worldwide invasion history of common ragweed, *Ambrosia artemisiifolia*. PLoS One.

[30] Gederaas L, Moen T, Skjelseth S, Larsen L (2012). Fremmede arter i Norge – med norsk svarteliste 2012.

[31] Gilbert B, Levine JM (2013). Plant invasions and extinction debts. P Natl Acad Sci USA.

[32] Gladieux P, Giraud T, Kiss L, Genton BJ, Jonot O, Shykoff JA (2011). Distinct invasion sources of common ragweed (*Ambrosia artemisiifolia*) in Eastern and Western Europe. Biol Invasions.

[33] Grime JP, Hodgson JG, Hunt R (1988). Comparative plant ecology. A functional approach to common British species.

[34] Hagenblad J, Hülskötter J, Acharya KP, Brunet J, Chabrerie O, Cousins SAO, Dar PA (2015). Data from Low genetic diversity despite multiple introductions of the invasive plant species *Impatiens glandulifera* in Europe. Dryad Digital Repository.

[35] Hardy OJ, Vekemans X (2002). SPAGEDi: a versatile computer program to analyse spatial genetic structure at the individual or population levels. Mol Ecol Notes.

[36] Hassel K, Såstad SM, Gunnarsson U, Söderström L (2005). Genetic variation and structure in the expanding moss *Pogonatum dentatum* (Polytrichaeceae) in its area of origin and in a recently colonized area. Am J Bot.

[37] Hauge M (1953). Noen ruderatblanter i Tønsberg. Blyttia.

[38] Henry P, Le Lay G, Goudet J, Guisan A, Jahodová S, Besnard G (2009). Reduced genetic diversity, increased isolation and multiple introductions of invasive giant hogweed in the western Swiss Alps. Mol Ecol.

[39] Huey RB, Gilchrist GW, Hendry AP, Sax DF, Stachowicz JJ, Gaines SD (2005). Using invasive species to study evolution. Species invasions: Insights to ecology, evolution and biogeography.

[40] Hulme PE, Bremner ET (2006). Assessing the impact of *Impatiens glandulifera* on riparian habitats: partitioning diversity components following species removal. J Appl Ecol.

[41] Hweitt GM (2008). Speciation, hybrid zones and phylogeography - or seeing genes in space and time. Mol Ecol.

[42] Jakobsson M, Rosenberg NA (2007). CLUMPP: a cluster matching and permutation program for dealing with label switching and multimodality in analysis of population structure. Bioinformatics.

[43] Jombart T, Devillard S, Balloux F (2010). Discriminant analysis of principal components: a new method for the analysis of genetically structured populations. BMC Genet.

[44] Kang M, Buckley YM, Lowe AJ (2007). Testing the role of genetic factors across multiple independent invasions of the shrub Scotch broom (*Cytisus scoparius*). Mol Ecol.

[45] Kliber A, Eckert CG (2005). Interaction between founder effect and selection during biological invasion in an aquatic plant. Evolution.

[46] Kollmann J, Bañuelos MJ (2004). Latitudinal trends in growth and phenology of the invasive alien plant *Impatiens glandulifera* (Balsaminaceae). Divers Distrib.

[47] Kurtto A (1996). *Impatiens glandulifera* (Balsaminaceae) as an ornamental and escape in Finland, with notes on the other Nordic countries. Act U Ups Symb Bot Ups.

[48] Lachmuth S, Durka W, Schurr FM (2010). The making of a rapid plant invader: genetic diversity and differentiation in the native and invaded range of *Senecio inaequidens*. Mol Ecol.

[49] Lavergne S, Molofsky J (2007). Increased genetic variation and evolutionary potential drive the success of an invasive grass. P Natl Acad Sci USA.

[50] Le Roux JJ, Brown GK, Byrne M, Ndlovu J, Richardson DM, Thompson GD (2011). Phylogeographic consequences of different introduction histories of invasive Australian Acacia species and *Paraserianthes lophantha* (Fabaceae) in South Africa. Divers Distrib.

[51] Le Roux J, Wieczorek A (2009). Molecular systematics and population genetics of biological invasions: towards a better understanding of invasive species management. Ann Appl Biol.

[52] Le Roux JJ, Wieczorek AM, Wright MG, Tran CT (2007). Super-genotype: global monoclonality defies the odds of nature. PLoS One.

[53] Lee CE (2002). Evolutionary genetics of invasive species. Trends Ecol Evol.

[54] Lee PLM, Patel RM, Conlan RS, Wainwright SJ, Hipkin CR (2004). Comparison of genetic diversities in native and alien populations of hoary mustard (*Hirschfeldia incana* [L.] Lagreze-Fossat). Int J Plant Sci.

[55] Lefèvre F, Fady B, Fallour-Rubio D, Ghosn D, Bariteau M (2004). Impact of founder population, drift and selection on the genetic diversity of a recently translocated tree population. Heredity.

[56] Levine JM, Vila M, D’Antonio CM, Dukes JS, Grigulis K, Lavorel S (2003). Mechanisms underlying the impacts of exotic plant invasions. P Roy Soc B Biol Sci.

[57] Lindley J: *Impatiens glanduligera*. Bot. Reg. N.S. 13, t.22; 1840.

[58] Mack RN, Simberloff D, Lonsdale WM, Evans H, Clout M, Bazzaz FA (2000). Biotic invasions: Causes, epidemiology, global consequences, and control. Ecol Appl.

[59] Mandák B, Hadincová V, Mahelka V, Wildová R (2013). European invasion of North American *Pinus strobus* at large and fine scales: High genetic diversity and fine-scale genetic clustering over time in the adventive range. PLoS One.

[60] Marrs RA, Sfprza R, Hufbauer RS (2008). When invasion increases population genetic structure: a study with *Centaurea diffusa*. Biol Invasions.

[61] Meekins JF, Ballard HE, McCarthy BC (2001). Genetic variation and molecular biogeography of a North American invasive plant species (*Alliaria petiolata*, Brassicaceae). Int J Plant Sci.

[62] Meirmans PG (2012). The trouble with isolation by distance. Mol Ecol.

[63] Mondini L, Noorani A, Pagnotta MA (2009). Assessing plant genetic diversity by molecular tools. Diversity.

[64] Nagy AM, Korpelainen H. Population genetics of Himalayan balsam (*Impatiens glandulifera*): comparison of native and introduced populations. Plant Ecol Div. 2014;1–5.

[65] Nei M (1972). Genetic distance between populations. Am Nat.

[66] Nei M, Maruyama T, Chakraborty R (1975). The bottleneck effect and genetic variability in populations. Evolution.

[67] Nordhagen R (1944). Bidrag till Norges flora. I. *Impatiens parviflora* DC., en ny ugrasplante på Vestlandet. Blyttia.

[68] Novak SJ, Mack RN (1993). Genetic variation in *Bromus tectorum* (Poaceae) - Comparison between Native and Introduced Populations. Heredity.

[69] Novak SJ, Mack RN, Sax DF, Stachowicz JJ, Gaines SD (2005). Genetic Bottlenecks in Alien Plant Species, Influence of Mating Systems and Introduction Dynamics. Species invasions: Insights to ecology, evolution and biogeography.

[70] Novak SJ, Mack RN, Soltis DE (1991). Genetic variation in *Bromus tectorum* (Poaceae): Population differentiation in its North American range. Am J Bot.

[71] Pairon M, Petitpierre B, Campbell M, Guisan A, Broennimann O, Baret PV (2010). Multiple introductions boosted genetic diversity in the invasive range of black cherry (*Prunus serotina*; Rosaceae). Ann Bot-London.

[72] Parker IM, Rodriguez J, Loik ME (2003). An evolutionary approach to understanding the biology of invasions: local adaptation and general‐purpose genotypes in the weed *Verbascum thapsus*. Conserv Biol.

[73] Pedersen A (1956). Rubiaceernes, Polygalaceernes, Linaceernes, Oxalidaceernes og Balsaminaceernes udbredelse i Danmark. Bot Tidsskr.

[74] Peery MZ, Kirby R, Reid BN, Stoelting R, Doucet-Beer E, Robinson S (2012). Reliability of genetic bottleneck tests for detecting recent population declines. Mol Ecol.

[75] Polunin O, Stainton A (1984). Flowers of the Himalaya.

[76] Prentis PJ, Sigg DP, Raghu S, Dhileepan K, Pavasovic A, Lowe AJ (2009). Understanding invasion history: genetic structure and diversity of two globally invasive plants and implications for their management. Divers Distrib.

[77] Pritchard JK, Stephens M, Donnelly P (2000). Inference of population structure using multilocus genotype data. Genetics.

[78] Provan JIM, Love HM, Maggs CAPRIMERNOTE (2006). Development of microsatellites for the invasive riparian plant *Impatiens glandulifera* (Himalayan balsam) using intersimple sequence repeat cloning. Mol Ecol Notes.

[79] Pyšek P, Prach K (1993). Plant invasions and the role of riparian habitats: a comparison of four species alien to central Europe. J Biogeogr.

[80] Pyšek P, Prach K (1995). Invasion dynamics of *Impatiens glandulifera* - a century of spreading reconstructed. Biol Conserv.

[81] R Core Team. R: A language and environment for statistical computing. R Foundation for Statistical Computing, Vienna, Austria: 2014. http://www.R-project.org.

[82] Raymond M, Rousset F (1995). GENEPOP (version 1.2): population genetics software for exact tests and ecumenicism. J Heredity.

[83] Rosenberg NA (2004). DISTRUCT: a program for the graphical display of population structure. Mol Ecol Notes.

[84] Rosenthal DM, Ramakrishnan AP, Cruzan MB (2008). Evidence for multiple sources of invasion and intraspecific hybridization in *Brachypodium sylvaticum* (Hudson) Beauv. in North America. Mol Ecol.

[85] Rousset F (1996). Equilibrium values of measures of population subdivision for stepwise mutation processes. Genetics.

[86] Rousset F (2008). genepop’007. a complete re-implementation of the genepop software for Windows and Linux. Mol Ecol Resour.

[87] Schaal BA, Gaskin JF, Caicedo AL (2003). The Wilhelmine W. Key 2002 Invitational Lecture. Phylogeography, haplotype trees, and invasive plant species. J Hered.

[88] Schlitzer R. Ocean Data View. http://odv.awi.de; 2015.

[89] Shirk RY, Hamrick JL, Zhang C, Qiang S (2014). Patterns of genetic diversity reveal multiple introductions and recurrent founder effects during range expansion in invasive populations of *Geranium carolinianum* (Geraniaceae). Heredity.

[90] Skálová H, Havlíčková V, Pyšek P (2012). Seedling traits, plasticity and local differentiation as strategies of invasive species of *Impatiens* in central Europe. Ann Bot-London.

[91] Slatkin M (1995). A measure of population subdivision based on microsatellite allele frequencies. Genetics.

[92] Squirrell J, Hollingsworth PM, Bateman RM, Dickson JH, Light MHS, MacConaill M (2001). Partitioning and diversity of nuclear and organelle markers in native and introduced populations of *Epipactis helleborine* (Orchidaceae). Am J Bot.

[93] Sun JH, Li ZC, Jewett DK, Britton KO, Ye WH, Ge XJ (2005). Genetic diversity of *Pueraria lobata* (kudzu) and closely related taxa as revealed by inter-simple sequence repeat analysis. Weed Res.

[94] Taylor DR, Keller SR (2007). Historical range expansion determines the phylogenetic diversity introduced during contemporary species invasion. Evolution.

[95] Thiébaut G. Non-indigenous aquatic and semiaquatic plant species in France. In: Dordrecht GF, editor. Biological invaders in inland waters: Profiles, distribution, and threats. Springer; 2007. p. 209–229.

[96] Toney JC, Rice PM, Forcella F (1998). Exotic plant records in the northwest United States 1950–1996: an ecological assessment. Northwest Sci.

[97] Tyler T, Johansson H, Olsson K-A, Sonesson M. Floran i Skåne. Arterna och deras utbredning: Lund: Lunds Botaniska Förening; 2007.

[98] Valliant MT, Mack RN, Novak SJ (2007). Introduction history and population genetics of teh invasive grass *Bromus tectorum* (Poaceae) in Canada. Am J Bot.

[99] Vitousek PM, DAntonio CM, Loope LL, Westbrooks R (1996). Biological invasions as global environmental change. Am Sci.

[100] Walker NF, Hulme PE, Hoelzel AR (2008). Population genetics of an invasive riparian species, *Impatiens glandulifera*. Plant Ecol.

[101] Ward SM, Gaskin JF, Wilson LM (2008). Ecological Genetics of Plant Invasion: What Do We Know?. Invasive Plant Sci Manag.

[102] Wares JP, Randall Hughes A, Grosberg RK, Sax DF, Stachowicz JJ, Gaines SD (2005). Mechanisms that Drive Evolutionary Change. Insights from Species Introductions and Invasions. Species invasions. Insights into ecology, evolution and biogeography.

[103] Warwick SI, Thompson BK, Black LD (1987). Genetic variation in Canadian and European populations of the colonizing weed species *Apera spica-venti*. New Phytol.

[104] Weber E (2003). Invasive plant species of the world: a reference guide to environmental weeds: Wallingford.

[105] Weir BS, Cockerham C (1996). Genetic data analysis II: Methods for discrete population genetic data. Inc.

[106] Williams DA, Overholt WA, Cuda JP, Hughes CR (2005). Chloroplast and microsatellite DNA diversities reveal the introduction history of Brazilian peppertree (*Schinus terebinthifolius*) in Florida. Mol Ecol.

[107] Wilson GA, Rannala B (2003). Bayesian inference of recent migration rates using multilocus genotypes. Genetics.

[108] Zhang YY, Zhang DY, Barrett SC (2010). Genetic uniformity characterizes the invasive spread of water hyacinth (*Eichhornia crassipes*), a clonal aquatic plant. Mol Ecol.

[109] Zhao J, Solís-Montero L, Lou A, Vallejo-Marín M (2013). Population structure and genetic diversity of native and invasive populations of *Solanum rostratum* (Solanaceae). PLoS One.

[110] Zybartaite LZJ, Jodinskiene M, Janssens SB, Paulauskas A, Kupcinskiene E (2011). RAPD analysis of genetic diversity among Lithuanian populations of *Impatiens glandulifera*. Žemdirbystė Agriculture.

