# Supplementary material for: Low genetic diversity despite multiple introductions of the invasive plant species Impatiens glandulifera in Europe
Source: BMC Genet. 2015 Aug 20;16:103. doi: 10.1186/s12863-015-0242-8 (PMC4546075; doi:10.1186/s12863-015-0242-8)

Full data set

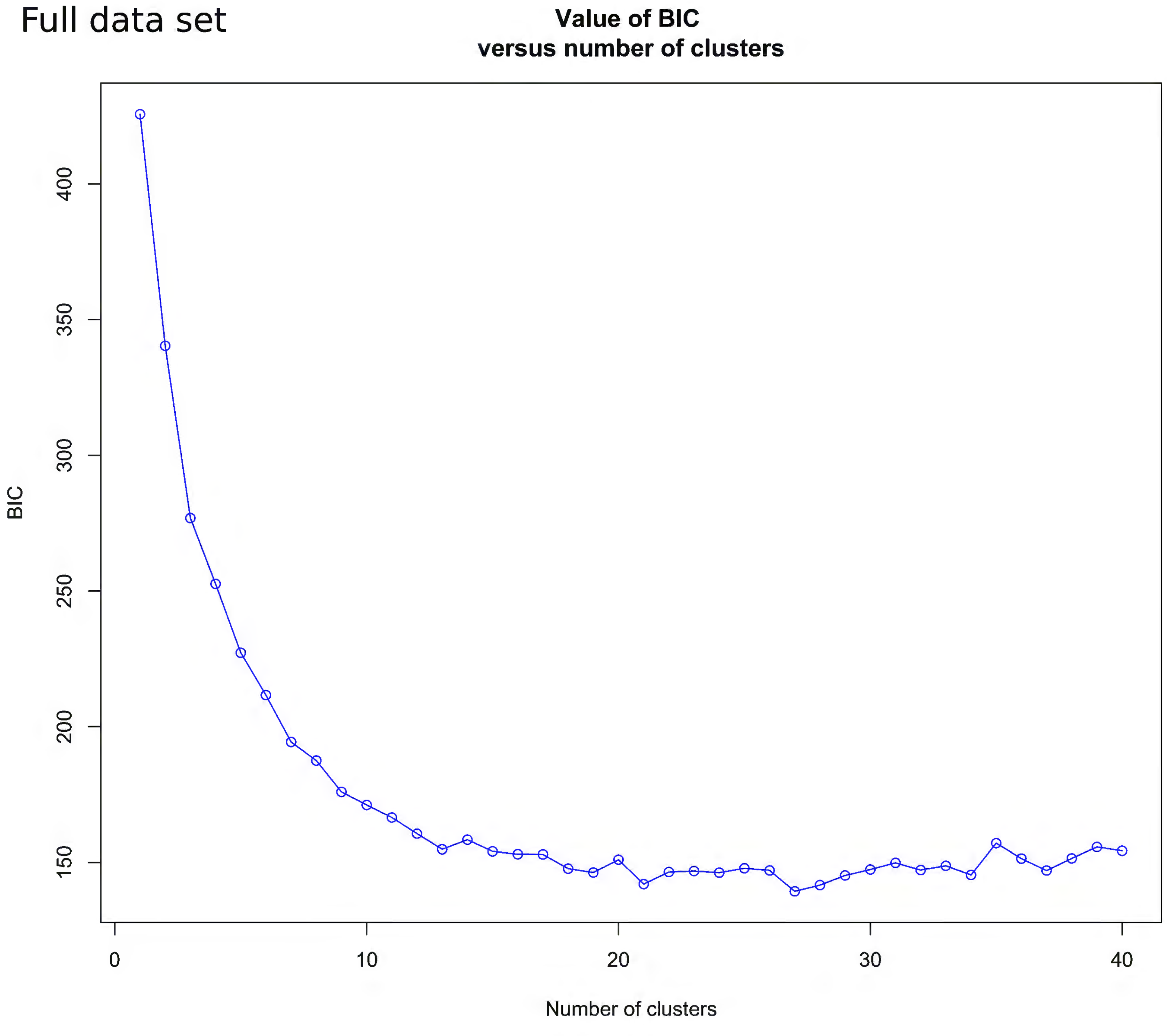

European data set

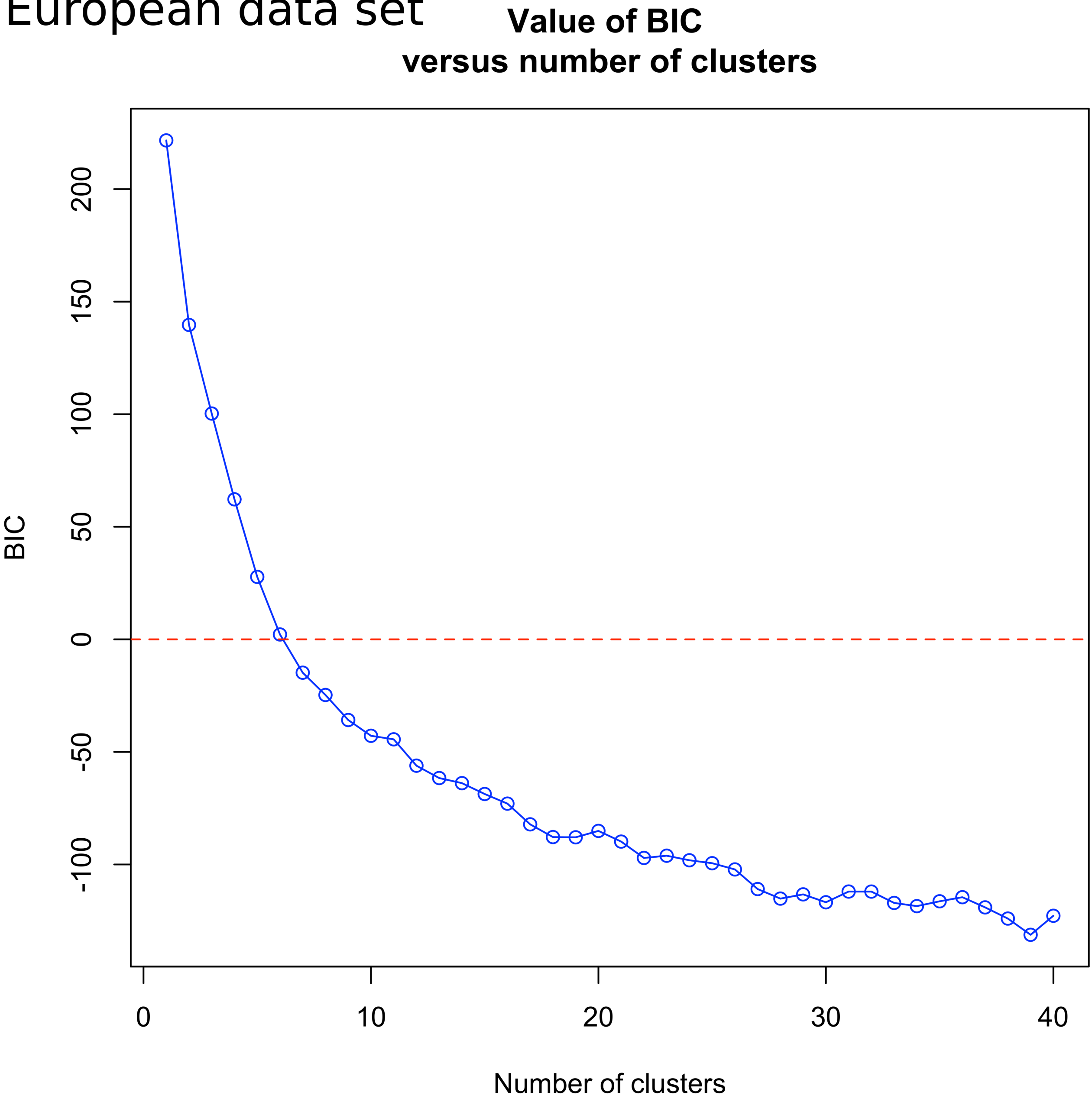

Kashmir data set

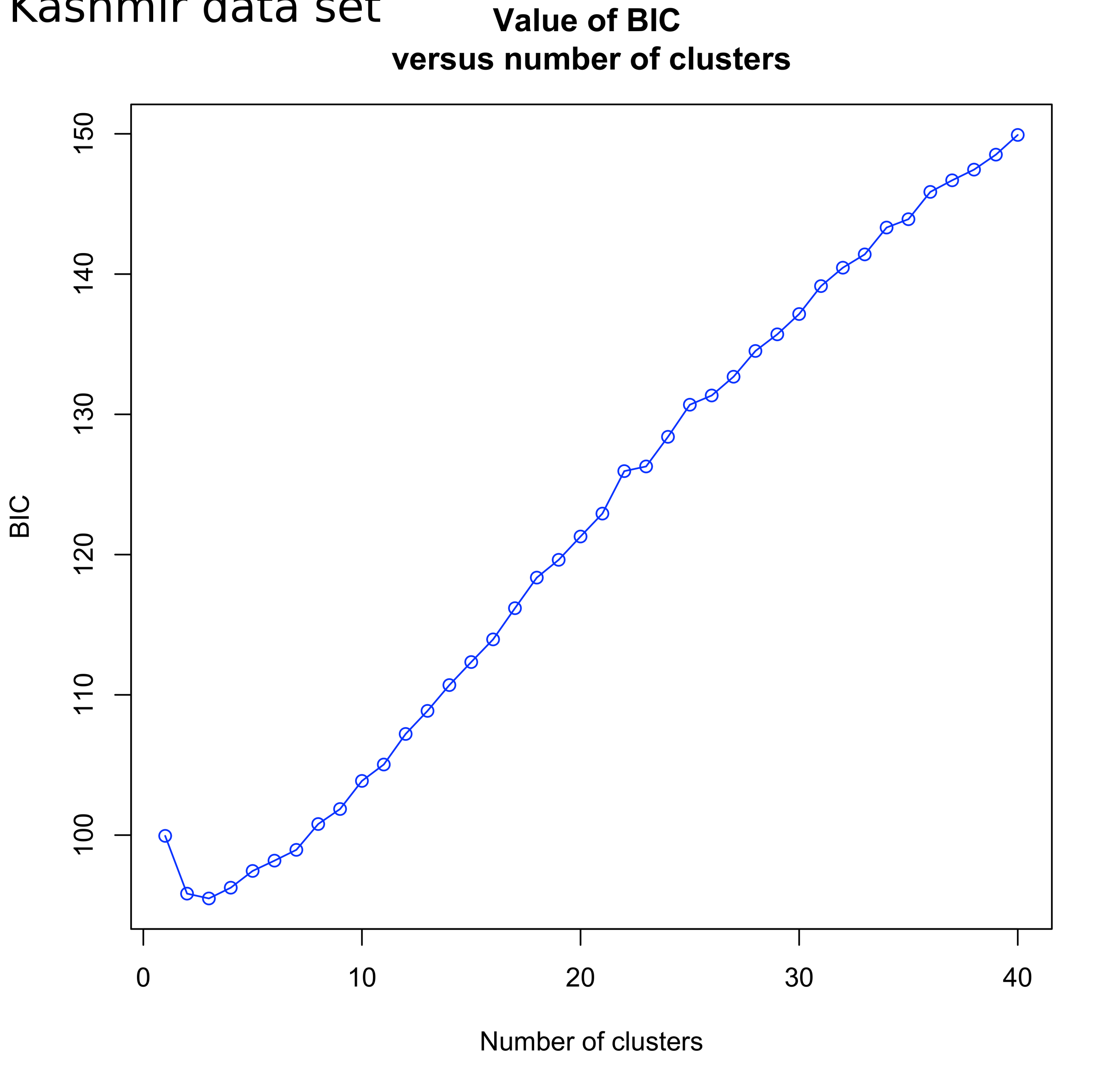

Supplement: Additional file 5: — BIC values for DAPC analyses of full, European and Kashmir data sets respectively. [file 12863_2015_242_MOESM5_ESM.pdf]
