# Supplementary figures and images for: Low genetic diversity despite multiple introductions of the invasive plant species Impatiens glandulifera in Europe
Source: BMC Genet. 2015 Aug 20;16:103. doi: 10.1186/s12863-015-0242-8 (PMC4546075; doi:10.1186/s12863-015-0242-8)

## Scenario 1

(Warning ! Time is not to scale.)

- N2c
- N1
- N2
- N3
- N2b

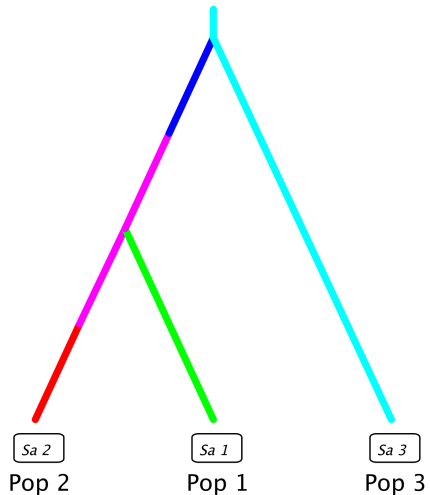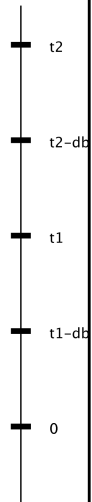

## Scenario 2

(Warning ! Time is not to scale.)

- N1b
- N1
- N2
- N3
- N2b

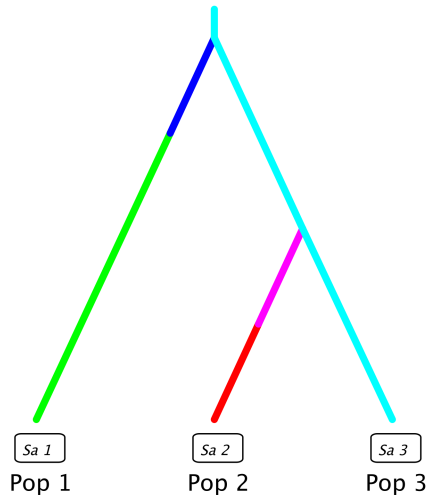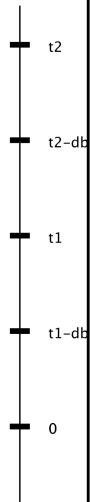

Supplement: Additional file 6: — Illustration of scenarios used in ABC analyses of population history with the recent history at the bottom of the panels and the past history at the top. Pop 1 = Southern European individuals, pop 2 = Northern European individuals, pop 3 = Kashmir individuals, with different colours denoting differences in population size. Scenario 1: Initial separation of all European individuals from Kashmir individuals at t2 followed by separation of the two European populations from each other at t1. Scenario 2: Initial separation of southern European individuals from northern European and Kashmir individuals at t2 followed by a later separation of the northern European individuals from the Kashmir ones at t1. [file 12863_2015_242_MOESM6_ESM.pdf]
