# Supplementary material for: Low genetic diversity despite multiple introductions of the invasive plant species Impatiens glandulifera in Europe
Source: BMC Genet. 2015 Aug 20;16:103. doi: 10.1186/s12863-015-0242-8 (PMC4546075; doi:10.1186/s12863-015-0242-8)
Supplement: Additional file 9: — Prior distribution of parameters used in ABC analysis. [file 12863_2015_242_MOESM9_ESM.pdf]

## Additional file 12

**Prior distribution of parameters used in ABC analysis. Population size parameters ($N_i$) are given in units of $N_e$ and time parameters ($t_i$) in units of generations. Gamma distribution shape parameter = 2. P: geometric distribution parameter, SNI: Single Nucleotide Insertion**

| Parameter | Distribution | Minimum | Maximum |
|---|---|---|---|
| **Demographic parameter priors** | | | |
| N1 | Uniform | 10 | 10000 |
| N1b | Uniform | 10 | 10000 |
| N2 | Uniform | 10 | 10000 |
| N2b | Uniform | 10 | 10000 |
| N2c | Uniform | 10 | 10000 |
| N3 | Uniform | 10 | 10000 |
| t1 | Uniform | 10 | 10000 |
| t2 | Uniform | 10 | 10000 |
| **Mutation model priors** | | | |
| Mean mutation rate | Uniform | $1 \times 10^{-4}$ | $1 \times 10^{-3}$ |
| Individual locus mutation rate | Gamma | $1 \times 10^{-5}$ | $1 \times 10^{-2}$ |
| Mean locus coefficient P | Uniform | 0.1 | 0.3 |
| Individual locus coefficient P | Gamma | $1 \times 10^{-2}$ | 0.9 |
| Mean SNI rate | Log-uniform | $1 \times 10^{-8}$ | $1 \times 10^{-4}$ |
| Individual locus SNI rate | Gamma | $1 \times 10^{-9}$ | $1 \times 10^{-3}$ |
